# The metabolic response to stress in critical illness: updated review on the pathophysiological mechanisms, consequences, and therapeutic implications

**DOI:** 10.1186/s13613-025-01588-z

**Published:** 2025-10-27

**Authors:** Raphael Mottale, Claire Dupuis, Sylwia Szklarzewska, Jean-Charles Preiser

**Affiliations:** 1 Department of Intensive Care, Université Libre de Bruxelles (ULB), Hôpital Universitaire de Bruxelles (H.U.B), CUB Hôpital Érasme, Brussels, Belgium; 2 Department of Internal Medicine, Université Libre de Bruxelles (ULB), Hôpital Universitaire de Bruxelles (H.U.B), CUB Hôpital Érasme, Brussels, Belgium; 3 Université Clermont Auvergne, Unité de Nutrition Humaine, INRAe, CRNH Auvergne, 63000 Clermont-Ferrand, France; 4 Department of Geriatric Medicine, Université Libre de Bruxelles (ULB), Hôpital Universitaire de Bruxelles (H.U.B), CUB Hôpital Érasme, Brussels, Belgium

**Keywords:** Anabolic resistance, Autophagy, Critical care, Early macronutrient restriction, Energy expenditure, Glycaemic control, Insulin sensitivity, Metabolomics, Post-intensive care syndrome

## Abstract

The understanding of the response to stress in critical illness has significantly improved in recent years. These adaptations unfold across acute, subacute, and chronic phases, with an early adaptive catabolic state, marked anabolic resistance, and a later transition toward recovery. The aim of this updated review is to summarize recent advancements focusing on pathophysiological changes in endocrine, immune, gut, and mitochondrial functions and their effects on the metabolic shift in energy production, using glycolysis and the utilization of lactate and ketones as alternative pathways to meet cellular energy demands. Advances in understanding key elements such as energy expenditure and autophagy have expanded our knowledge. Furthermore, there is increased interest in the consequences of an intense and prolonged stress response, which can lead to ICU-acquired weakness (ICU AW) and post-intensive care syndrome. Recent evidence indicates that higher protein strategies generally do not improve survival or functional recovery and may signal harm in patients with renal dysfunction, supporting cautious, phase-appropriate protein dosing rather than routine high targets. New concepts, such as chronic critical illness (CCI) and persistent inflammation, immunosuppression, and catabolism syndrome (PICS), have also emerged to characterize prolonged stress responses. For glycaemic management, intensive control offers no outcome benefit and increases hypoglycaemia risk; moderate targets are preferred. Parallel advancements in monitoring techniques, such as indirect calorimetry and body composition analysis, have improved the assessment of the consequences of the metabolic changes. Metabolomics has offered deeper characterisation of the metabolic response to stress and nutrition, highlighting key metabolic pathways and potential therapeutic targets. Integrating biomarkers and metabolomics to define clinical endotypes may help time the transition from catabolic to anabolic strategies and personalize nutrition and pharmacologic support at the bedside. New therapeutic avenues have emerged or are under investigation, including glycaemic control, nutritional strategies, and some specific interventions targeting key components of the metabolic response. In this context, we present a narrative review of the literature with a focus on the clinical consequences of the pathophysiological and metabolic response to stress, alongside therapeutic implications and future perspectives.

**Supplementary Information:**

The online version contains supplementary material available at 10.1186/s13613-025-01588-z.

## Introduction

Metabolism is a finely tuned system, balancing the storage of energy and synthesis molecules (anabolism) with energy release (catabolism). During critical illness, the metabolic response to stress (MRS) is triggered by various stimuli and described in three phases: acute, subacute, and chronic. These phases involve endocrine and immune-inflammatory responses, leading to significant changes in cellular and mitochondrial functions.

Unlike physiological conditions, the transition from catabolism to anabolism is impaired due to “anabolic resistance”, complicating metabolic recovery. Hence, the detection of the post-injury phases represent a major challenge in metabolic care. Inappropriate nutritional intakes can result in ICU-acquired weakness and chronic critical illness, leading to prolonged hospital stays, delayed recovery, and increased morbidity [1–5].

Since the last major review on MRS [6], advancements in metabolomics [7] and assessment in body composition [5, 8, 9] have improved its characterisation, revealing its complex interactions with nutrition. A paradigm shift is gradually taking place around the concepts of energy targets throughout MRS phases. The previous concept of “more is better”, advocating for 100% expenditure matching from the acute phase and high protein intake to counteract muscle loss, is now challenged by recent clinical data. [8, 10–12]. Excessive early macronutrient is potentially harmful [1, 4, 13–17]. The mechanisms underlying the detrimental effects of high nutrient provision are partially understood, and include overfeeding, refeeding syndrome, dysregulated mitochondrial function, suppression of repair pathways (autophagy) and ketogenesis, as suggested by both preclinical and clinical studies [2, 14, 15, 18–20].

This review provides a comprehensive yet focused overview of the metabolic response to stress (MRS) in critical illness, with the aim of clarifying its key mechanisms and clinical relevance. The article is structured into three main sections, each addressing a distinct but interconnected dimension of the MRS: (1) The first section, Pathophysiology, describes the initial systemic and cellular responses triggered by acute stress. (2) The second section, Metabolic Consequences, explores how these pathophysiological processes alter substrate metabolism—affecting glucose, lipids, amino acids, and intermediary energy pathways such as lactate and ketone bodies. It also introduces the concept of distinct metabolic phases that may be reflected in clinical and molecular signatures. (3) The third section, Clinical Consequences and Therapeutic Implications, focuses on the observed effects of metabolic stress. It discusses how insights from metabolomics and advanced monitoring tools (e.g., indirect calorimetry, body composition analysis) may inform personalized nutritional and pharmacologic strategies to support recovery. The principal advances related to MRS are summarized in Table 1.

**Table 1:** What’s new ?

- Better characterisation of the metabolic response to stress with three distinct metabolic phases in ICU patients	
- Early macronutrient restriction, including energy and proteins, during the acute phase is associated with better outcomes than full feeding	
- Micronutrient supply should be considered in medical situations at risk of depletion	
- Technical improvements of bedside tools to determine energy expenditure and body composition	
- Biomarkers and metabolomics as tools to identify the transition between the acute catabolic phase and the later anabolic phase	

## Search strategy

The search strategy is detailed in the supplementary data.

## Pathophysiology

**Fig. 1:**
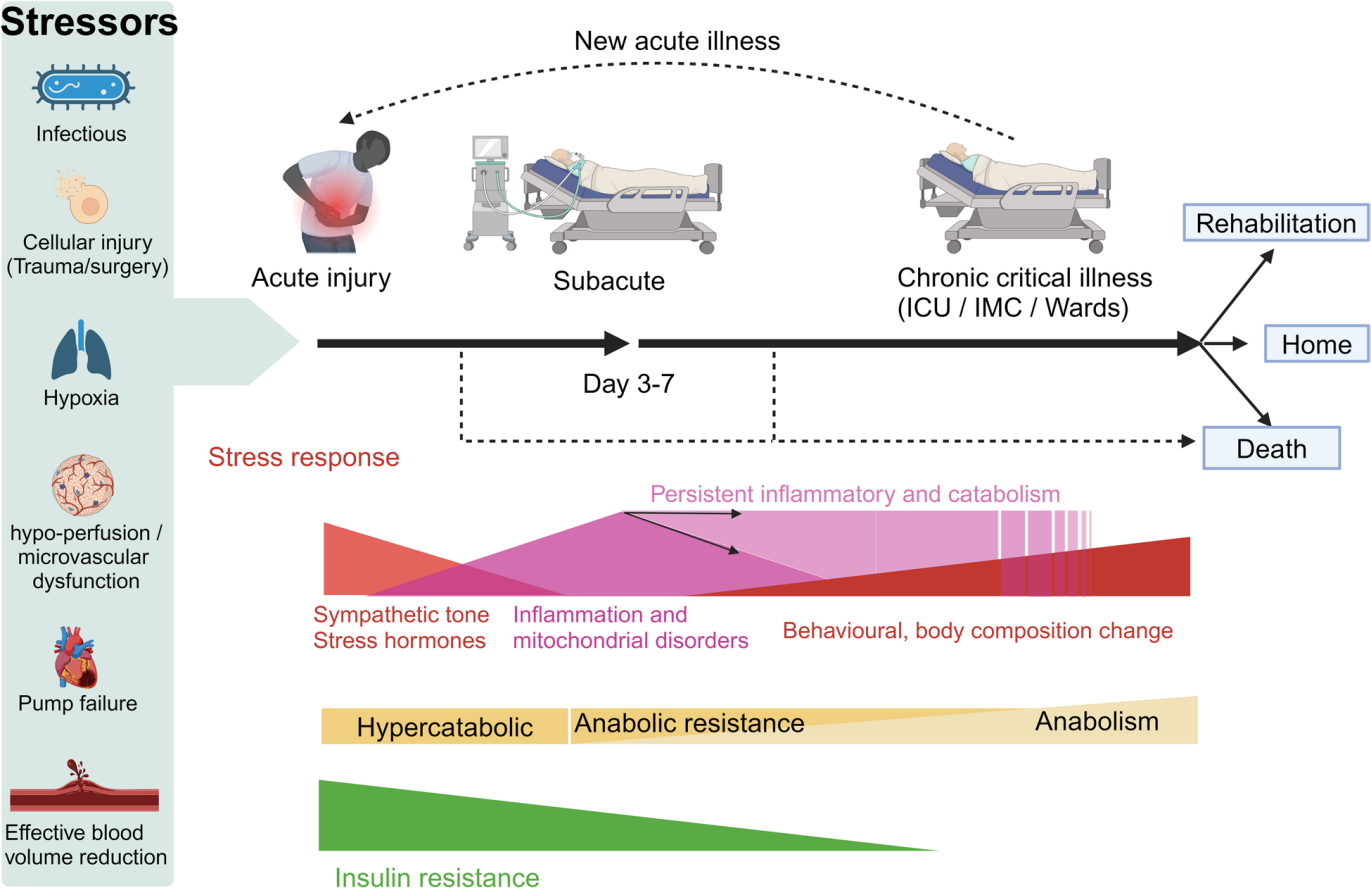
Time course of acute illness. Intense physiological stressors listed in the left blue rectangle trigger a reflex response of the autonomic nervous system and hormonal systems (acute phase). This phase is followed by systemic amplification or development of inflammation, activation of the immune system, and mitochondrial dysfunction (subacute phase). The patient then transitions into a chronic phase characterised mainly by behavioural disturbances and changes in body composition.During the acute and subacute phases, the body undergoes catabolism with some anabolic resistance, potentially correlated with the degree of insulin resistance. A patient's progression can vary from complete recovery to death. Several acute episodes may occur during hospitalisation. After the subacute phase, a process of resolution of inflammation is initiated except in the specific situation in which inflammation and catabolism persist (persistent inflammatory and catabolism syndrome, PICS). *ICU* intensive care unit. *IMC* intermediate care. Created with BioRender.com

The stress-induced metabolic response, previously described by Cannon and Selye as a single fight-or-flight response, is now understood to be a complex, interconnected process involving different mechanisms and unfolding over three consecutive phases: acute, subacute, and chronic (Fig. 1).

### Acute phase (upper part of Fig. 2)

**Fig. 2:**
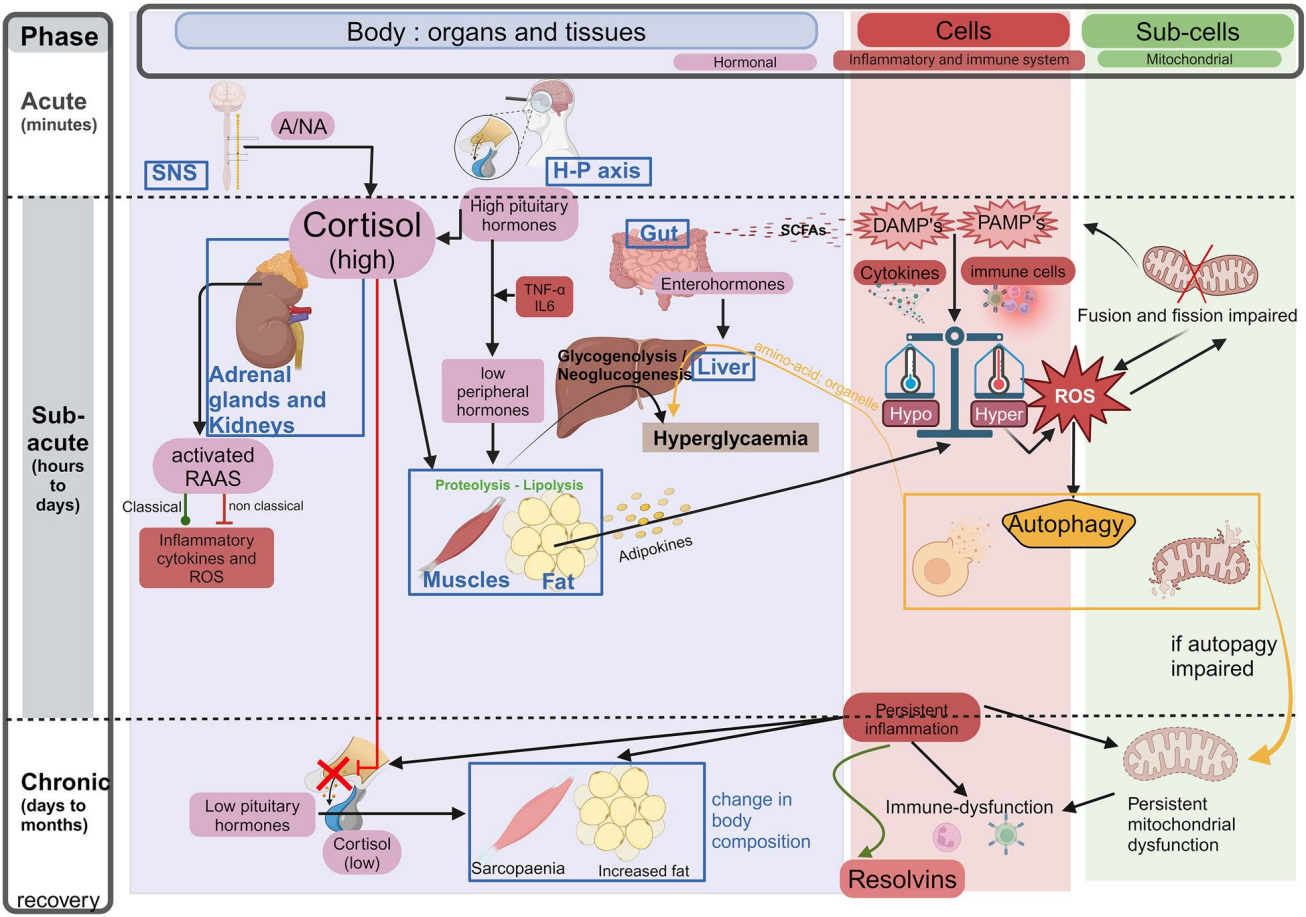
The complex puzzle of the metabolic response to stress. On the left side, the duration of the different phases (acute, subacute, and chronic) is represented. At the top, the various players in the metabolic response are listed, categorised by their involvement with organs (blue), cells (red), and subcellular levels (green). In the box labelled " Body: organs and tissues (blue), the main players include the sympathetic nervous system (SNS), the hypothalamic-pituitary axis, the adrenal gland, the kidneys, the gut, muscles and adipose tissue, as well as the liver. Most interactions between these players are mediated by hormones (pink). The primary consequence is the liver's production of glucose, which serves as the main energy source. In the box labelled "Cells" (red), the focus is on the generation of inflammation through the involvement of the immune system, whether it be of infectious origin (PAMPs) or related to cellular damage (DAMPs). In the box labelled "Sub-cells" (green), mitochondrial dysfunction and the generation of oxidative stress are shown. The primary mechanism at play is autophagy. Black arrows represent the consequences and interactions between different players. Red arrows indicate a negative effect (or feedback loop), while green arrows indicate a positive effect or resolution. *A/NA* adrenaline/noradrenaline (catecholamines), *DAMP* damage-associated molecular pattern, *H-P axis* hypothalamo-pituitary axis, *PAMP* pathogen associated molecular pattern, *RAAS* renin angiotensin aldosterone system, *ROS* reactive oxygen species, *SCFAs* short-chain fatty acids, *SNS* sympathetic nervous system. Created with BioRender.com

This phase is initiated by sympathetic system activation, stimulated by baroreceptors and inflammatory mediators, leading to catecholamine release via postganglionic neurons. It subsequently activates the hypothalamic–pituitary axis and releases adrenocorticotropic hormone (ACTH), thyroid-stimulating hormone (TSH), growth hormone (GH), prolactin (PRL), gonadotropins (LH, FSH), and arginine vasopressin (AVP) [6, 21]. AVP plays a key role by promoting vasoconstriction via V1a receptors and enhancing renal water reabsorption through V2 receptors [22].

### Subacute phase (middle part of Fig. 2)

#### Hormonal and neuro-endocrine response

The ongoing activation of the hypothalamic-pituitary axis, induces changes in most circulating hormones from peripheral glands either decrease (e.g., insulin-like growth factor, testosterone) or convert to inactive forms (e.g., reverseT3) shortly after production [21]. This decrease in release of peripheral hormones despite an increase in the stimulus is mainly attributed to the abundance of inflammatory cytokines released, tissue hypoxia, elevated cortisol levels, and commonly used medications in the ICU [21, 23, 24]. These hormonal alterations, typically reported as 'peripheral growth hormone resistance' and 'non-thyroidal illness,' may prevent the detrimental metabolic effects induced by the GH or the thyroid hormones such as increased mitochondrial oxygen consumption and stimulation of thermogenesis, which could exacerbate cellular stress [21, 23, 25].

In contrast to other peripheral hormones, free cortisol levels increase not primarily due to increased production by ATCH stimulation, but rather due to delay in inactivation, and decrease in cortisol-binding proteins. Cortisol promotes catabolism in muscle and adipose tissue to provide substrates for energy-consuming organs [21, 26]. After an initial rise, ACTH, TSH, and AVP levels decline due to negative feedback mechanisms, mainly driven by elevated circulating cortisol levels and altered hypothalamic signaling [23].

The renin–angiotensin–aldosterone system is also activated to facilitate sodium and water reabsorption. Classic and non-classic components have distinct physiological roles through the production of angiotensin II-III-IV or angiotensin 1–9 and 1–7, respectively [27]. The classic system increases pro-inflammatory markers, reactive oxygen species (ROS), and apoptosis, whereas the non-classic system is believed to have an immunomodulatory effect and increases pulmonary perfusion as suggested by in vitro and animal studies [27]. Elevated aldosterone levels may also contribute to insulin resistance and increased free fatty acids [28].

#### Inflammatory and immune pathways

Recent insights into the inflammatory response unraveled the metabolic effects of mediators such as endogenous alarmins (or damage-associated molecular patterns (DAMPs) during cellular lysis and exogenous pathogen-associated molecular patterns (PAMPs) in the presence of infectious disease). DAMPs and PAMPs are recognised by various immune cell receptors (TLRs (Toll-like receptors), lectin receptors, nucleotide-binding oligomerization domain). This triggers a complex immune and inflammatory response characterised by activation of leucocytes, complement, and coagulation leading to a pro-inflammatory response, and concomitantly by an anti-inflammatory response associated with immune cell alterations through apoptosis and cellular regulation mechanisms [29–31]. These phenomena have been associated with different subphenotypes termed hyperinflammatory and hypoinflammatory, in patients with sepsis or acute respiratory distress syndrome (ARDS) [32]. The hyperinflammatory subphenotype is marked by elevated levels of circulating proinflammatory cytokines and higher mortality, while the hypoinflammatory phenotype is associated with lower inflammatory markers and better outcomes. Recent transcriptomic and metagenomic analyses have confirmed that these phenotypes also differ in immune response pathways, with upregulation of innate immune signaling and metabolic pathways (e.g., glycolysis, oxidative phosphorylation) in the hyperinflammatory group, and enhanced adaptive immunity and T cell–related gene expression in the hypoinflammatory group [33].

In the past decade, adipokines, secreted by white (WAT) and brown (BAT) adipose tissues, have emerged as novel mediators in the inflammatory response and act as endocrine regulators. In human observational studies and animal experiments, WAT releases pro-inflammatory adipokines like leptin and resistin, and anti-inflammatory ones such as adiponectin and omentin-1, impacting immune regulation and metabolism [34, 35]. Meanwhile, BAT produces neuregulin-4, fibroblast growth factor-21, and myostatin, which enhance thermogenesis and vascular remodeling, but negatively regulate skeletal muscle growth [36]. Additionally, skeletal muscle secretes myokines like irisin, promoting energy expenditure, and myostatin [35].

#### Gut dysfunction

The gastrointestinal system may contribute to the MSR. Firstly, stress‑induced splanchnic vasoconstriction and hypovolemia reduce mucosal perfusion, producing epithelial hypoxia and apoptosis, and priming the gut for ischemia‑reperfusion injury. Concomitant disruption of tight junction proteins increases paracellular leak, while enterocyte loss slows renewal and widens mucosal breaches. These structural failures favour dysbiosis with collapse of short‑chain‑fatty‑acid (SCFAs) producing taxa, curtailing SCFA‑mediated epithelial nutrition and immunomodulation [37]. In parallel, medication use and the hospital environment exacerbate dysbiosis, further reducing SCFA production, increasing bacterial translocation, and potentially amplifying inflammatory pathways of the MSR [37, 38]. Loss of immunomodulation, dysbiosis, and gut barrier failure lead to the translocation of microbial components via PAMPs, activating TLRs and triggering a systemic cytokine surge that promotes insulin resistance, protein catabolism, and mitochondrial dysfunction [39, 40]. Secondly, alterations in enterohormone levels (glucagon-like peptide-1, peptide YY, and ghrelin) in critically ill patients may affect gastroparesis, glycaemic control, nutrient absorption and energy utilization [41, 42].

#### Mitochondrial dysfunction and autophagy

Under physiological conditions, mitochondrial fusion maintains function by mixing contents, while fission allows segregation of damaged components. During acute injury, this balance is disrupted where fusion is impaired and fission prevails, leading to fragmentation, reduced function, and the release of mitochondrial DAMPs such as N-formyl peptides, followed by degradation via mitophagy [43, 44]. A metabolomic study shows that circulating N-formylmethionine, a mitochondrial-derived molecule, can be used as a biomarker of incomplete mitochondrial fatty acid oxidation, reflecting a bioenergetic failure [44]. Mitochondrial dysfunction is directly associated with reduced ATP (Adenosine Triphosphate) production and poor outcomes in sepsis [45].

In healthy states, ROS are byproducts of oxidative phosphorylation, tightly regulated by antioxidants.. In stress conditions, mitochondrial damage and electron leakage increase ROS production, leading to oxidative injury [46], which in turn promotes autophagy via adenosine monophosphate-activated protein kinase (AMPK) activation and mammalian target of rapamycin (mTOR) inhibition[47]. mTOR is a central metabolic regulator that integrates nutrient, energy, and growth factor signals to control cell growth and metabolism [48]. Autophagy appears to be a key mechanism in the survival of critically ill patients. It promotes degradation of damaged organelles and recycles substrates under energetic stress. It is activated by nutrient deprivation, hypoxia, and ROS. Insufficient autophagy, evidenced by accumulated organelle damage in patient biopsies, is associated with higher mortality in pre-clinical and clinical studies [2, 49].

There appears to be a close regulatory relationship between autophagy and metabolism where excess nutrient intake may inhibit autophagy via insulin/mTOR signaling while insulin resistance in the acute setting may promote autophagy by reduced activation of mTOR and overactivation of forkhead box O proteins [2, 48, 49].

### Chronic phase to recovery (lower part Fig. 2)

Some patients rapidly recover after the initial phases, while others experience prolonged ICU stays and chronic symptoms. These cases are described using various terms, such as post intensive care symptoms, chronic critical illness (CCI), persistent critical illness, and persistent inflammation, immunosuppression, and catabolism syndrome (PICS), which share overlapping features but each have their own specific focus [50].

The pathophysiology explaining why some patients improve rapidly while others develop persistent inflammation and catabolism remains poorly understood.

Anabolic recovery is promoted by interconnected mechanisms.

Specialized Pro-resolving Mediators (SPMs), derived from essential fatty acids, are rapidly produced following cellular injury and play a pivotal role in resolving inflammation. These mediators inhibit leukocyte chemotaxis, reduce adhesion between leukocytes and the endothelium, and decrease the production of eicosanoids and inflammatory cytokines—effects demonstrated mostly in preclinical models [55, 56] and associated with survival in observational studies [51].

Insulin sensitivity progressively improves as pro-catabolic hormones decline and inflammation resolves, restoring Insulin receptor substrate-1 phosphorylation and reactivating the PI3K pathway [48, 49]. This lead to mTOR activation, also driven by decreased AMPK activity, the reactivation of the PI3K pathway, and the recirculation of key amino acids such as leucine [48, 49, 52]. Furthermore, mitochondria regain their functional capacity, and levels of ROS decrease [53].

Persistent inflammation and catabolism may be sustained or exacerbated by chronic pressure ulcers, microbiota impairment [54], or continuous renin–angiotensin–aldosterone system activation [28]. The frequently reported immunosuppression may be linked to the persistence of mitochondrial dysfunction [43], alteration in cytokines profiles [55], and persistence in myeloid derived suppressor cells [56].

Reduced plasma levels of pituitary factors and peripheral hormones -due to persistent inflammation, circadian disruption and feedback inhibition by the initial increase in cortisol- contribute to the chronic critical illness phenotype, manifesting as residual vasoplegia, fatigue, encephalopathy, electrolyte imbalances, ICU-AW, and diaphragm dysfunction [21, 57].

## Metabolic consequences

### From the acute to the subacute phase (see Fig. 3)

**Fig. 3:**
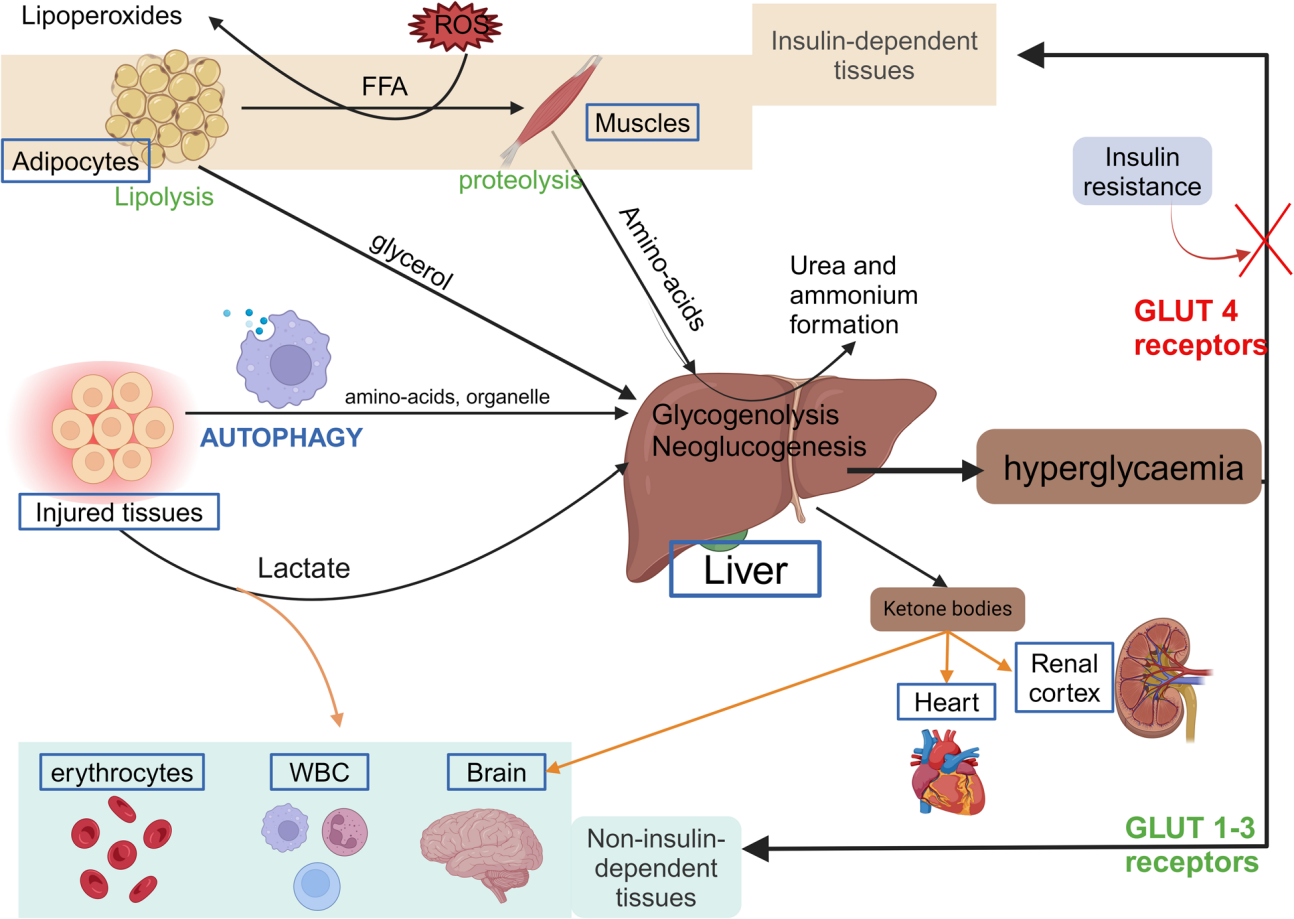
Metabolic consequences during acute illness. This figure synthesises the metabolic changes related to stress. Glucose is the fastest energy source generated by the liver through amino acids, free fatty acids (FFAs), glycerol, lactate, and organelles produced by catabolism and autophagy. Glucose feeds non-insulin dependent tissues by passive glucose uptake through GLUT 1–3 receptors, while insulin-dependent tissues cannot uptake glucose due to downregulation of GLUT-4 receptors. Other metabolic pathways, such as those involving lactate and ketone bodies, are highlighted in orange and provide an alternative energy source. *FFA* free fatty acids. *ROS* reactive oxygen species*, WBC* White blood cells*. Created with BioRender.com*

Glucose becomes the preferred energy substrate, and a metabolic shift occurs in energy production, from mitochondrial β-oxidation of fatty acids toward glycolysis, branched-chain amino acid metabolism, and activation of the pentose phosphate pathway [6, 44]. This shift reflects mitochondrial dysfunction and greater reliance on glycolysis, as shown by a metabolomic signature of elevated N-formylmethionine, lactate, and acylcarnitines, indicating impaired fatty acid oxidation and oxidative stress [44].

The liver maintains glucose availability via rapid glycogenolysis and gluconeogenesis using amino acids and glycerol derived from muscle catabolism and adipose tissue.

Proteins breakdown increases up to threefold during stress, producing nitrogen waste as urea and ammonium [58].

Although lipolysis is enhanced during critical illness to provide glycerol for hepatic gluconeogenesis, mitochondrial dysfunction and limited oxygen delivery impair β-oxidation. This mismatch leads to the accumulation of free fatty acids, which contributes to insulin resistance and lipotoxicity through lipid peroxidation. [59]. Cholesterol levels decrease significantly in sepsis due to reduced hepatic synthesis and increased lipoprotein consumption—a pattern associated with higher mortality, ascholesterol is essential in cell membrane integrity, cellular signalling and asa precursor for many hormones, including cortisol [60].

All of the MRS mechanisms contribute to “stress hyperglycaemia”. Insulin resistance, driven by counter-regulatory hormones and inflammation, plays a key role in this process [61]. Peripheral insulin resistance allows glucose to be redirected to vital organs by upregulating GLUT-1 and -3 in erythrocytes, immune cells, and brain, while downregulating GLUT-4 in skeletal muscle and adipose tissue[62].

Recently, the lactate and ketone body pathways have been shown to be important alternative energy pathways. Lactate metabolism, by oxidation [63], plays a pivotal role in energy backup especially in tissues with low mitochondrial concentrations, such as the renal medulla, brain, erythrocytes, and leucocytes [61]. lactate is rapidly converted to pyruvate via lactate dehydrogenase (LDH) and directly enters the Krebs cycle, providing an ATP-efficient fuel source within the framework of the “lactate shuttle”. [64, 65]. This shuttle, facilitated by monocarboxylate transporters (MCT1 and MCT4), enables lactate to act as a dynamic energy carrier between producing and consuming cells, adapting to metabolic stress and hypoxia [66]. Its signaling capabilities, acting as an autocrine, paracrine, and endocrine-like agent, are now recognised, earning it the term “lactormone” [64, 65]. In addition to these metabolic roles, lactate has significant immunomodulatory properties, influencing immune cell activity through several signalling pathways. Lactate modulates macrophage polarization by promoting M2 (anti-inflammatory) macrophages [64, 67]. This regulation supports wound healing and anti-inflammatory responses, whereas M1 (pro-inflammatory) macrophages rely more on glycolysis. Furthermore, lactate regulates neutrophil activity by promoting NETosis and can suppress pro-inflammatory cytokine production in other immune cells​ [65, 67]​.

Ketone bodies (KBs) represent an additional potential source of energy while the organism is fasting or under acute stress.Their synthesis begins with the breakdown of fatty acids into acetyl-CoA, which is converted into KBs when the tricarboxylic acid cycle is overwhelmed. They are the preferred energy source for the heart and renal cortex, and can provide up to 75% of the energy consumed by the brain. KBs, particularly β-hydroxybutyrate (BHB), play a crucial role in critical illness by activating protective cellular pathways. In animals models, BHB enhances autophagy by inhibiting histone deacetylases, promoting the clearance of damaged organelles [19, 68–70], suppresses the NLRP3 inflammasome (NOD-like receptor family, pyrin domain containing 3), reducing pro-inflammatory cytokines [68, 69], and activates Nrf2 protecting against oxidative damage, and promoting mitochondrial biogenesis, crucial for cellular recovery​ [69, 70]. BHB supports muscle regeneration by increasing Insulin-like Growth Factor One levels and facilitating mTOR-mediated protein synthesis [68, 70].

### Chronic phase to recovery

After acute illness, a transition from a catabolic to an anabolic state typically occurs, marking the onset of recovery. The restoration of insulin sensitivity during the recovery phase of critical illness shifts metabolic priorities, reducing glucose dependency and favoring the use of lipids and proteins [6]. Lipids regain importance as energy substrate through efficient β-oxidation, supported by restored mitochondrial function. Proteins and amino acids also play a central role during this phase; with reduced gluconeogenesis, amino acids are redirected towards anabolic processes. Activation of the mTOR pathway, driven by insulin signaling and key amino acids such as leucine, promotes protein synthesis and tissue repair [48, 49, 52, 71].

However, in certain cases, a state of persistent inflammation, immune dysfunction, and chronic catabolism may develop, known as PICS. Glucose remains the preferential energy substrate, accompanied by ongoing proteolysis and lipolysis [50].

### A metabolic signature to characterize the patient's state?

To date, metabolic characterisation of these patients remains challenging. A combination of methods could help estimate the patient's metabolic state. Changes in nitrogen balance or a raised urea-to-creatinine ratio can be used to evaluate catabolism, although their interpretation may be confounded in cases of renal dysfunction or severe muscle atrophy [5, 72, 73]. The development of tools to measure insulin resistance using continuous glucose monitoring methods represents a promising approach, as increasing insulin resistance in ICU patients contributes to hyperglycaemia and requires higher insulin doses to maintain glycaemic control despite similar nutritional intake [74, 75]. Finally, the use of biomarkers and metabolomics enables the identification of key markers of metabolic states and holds promise as the next generation of point-of-care tools. For example, mitochondrial dysfunction can be assessed by measuring N-formylmethionine, short‑chain acylcarnitines, and NAD(P)‑related metabolites, which reflect impaired oxidative phosphorylation and incomplete fatty acid oxidation [44, 76]. Autophagy activity may be inferred from tissue-level proteomic data. In ICU patients, accumulation of p62 (a cargo adaptor protein normally degraded during autophagy) and decreased LC3-II/LC3-I ratios (reflecting impaired autophagosome formation or turnover) in liver and muscle biopsies have been associated with insufficient autophagic activation, leading to the accumulation of damaged mitochondria and endoplasmic reticulum [49].

Changes in amino acid profiles, identified through metabolomic and proteomic analyses, provide functional insight into the metabolic phases of critical illness. In septic patients, Chen et al. identified consistent dysregulation of amino acid pathways involved in immune and mitochondrial regulation, including arginine biosynthesis, glutamine depletion, and tryptophan conversion into kynurenine. The accumulation of kynurenine, in particular, was associated with immunosuppression and redox imbalance, suggesting a transition to a persistent catabolic or inflammatory state [52]. In parallel, alterations in circulating amino acids; especially arginine, proline, and glutamate, during enteral and parenteral nutrition may reflect how substrate delivery modulates intermediary metabolism and help define the patient's underlying metabolic trajectory [73].

Lipidomic signatures evolve across phases of critical illness and reflect both energy substrate shifts and structural cell alterations. In untargeted plasma lipidomics analyses of ICU patients, reduced levels of phosphatidylcholines, a key components of mitochondrial and cellular membranes, have been reported, potentially impairing membrane integrity and mitochondrial function under oxidative stress. Concurrent accumulation of medium- and long-chain acylcarnitines suggests incomplete β-oxidation and mitochondrial substrate overload, interfering with ATP production and promoting lipotoxicity [7].

Markers of intestinal dysfunction have also been identified through metabolomic profiling. Reduced plasma citrulline, a non-protein amino acid synthesized exclusively by enterocytes, is a validated biomarker of enterocyte functional mass and impaired absorptive capacity [77]. In parallel, altered tryptophan metabolism, including elevated levels of kynurenine and indole-derived microbial metabolites, reflects intestinal barrier dysfunction and dysbiosis [73].

Muscle-specific transcriptomic signatures in long-term ICU survivors reveal sustained downregulation of genes involved in mitochondrial respiration, oxidative phosphorylation and myofibrillar structure, which reflect bioenergetic failure and a prolonged catabolic state resistant to anabolic transition [78].

Recently, targeted metabolomic profiling of patients with septic and cardiogenic shock identified three distinct metabolic clusters at ICU admission, each associated with differing mortality risks and metabolic patterns [79]. Cluster 1, associated with the highest 28-day mortality (54%), exhibited elevated levels of biogenic amines, sugars, and sphingolipids—suggesting intense metabolic activation, impaired mitochondrial function, and a hypercatabolic state. Cluster 2, despite lower initial clinical severity, had a high mortality rate (38%) and was characterized by increased glycerophospholipids and sphingolipids but lacked the energy-related metabolites observed in Cluster 1, consistent with an energetically deficient or maladaptive profile. Cluster 3, with the lowest mortality (9%), showed globally reduced metabolite levels across most pathways, reflecting a more regulated, metabolically adaptive response potentially compatible with anabolic recovery.

Similar observations have been made in sepsis [44, 52, 80, 81] and acute respiratory failure [76] -focused multi-omics studies.

## Clinical consequences and therapeutic implications

**Fig. 4:**
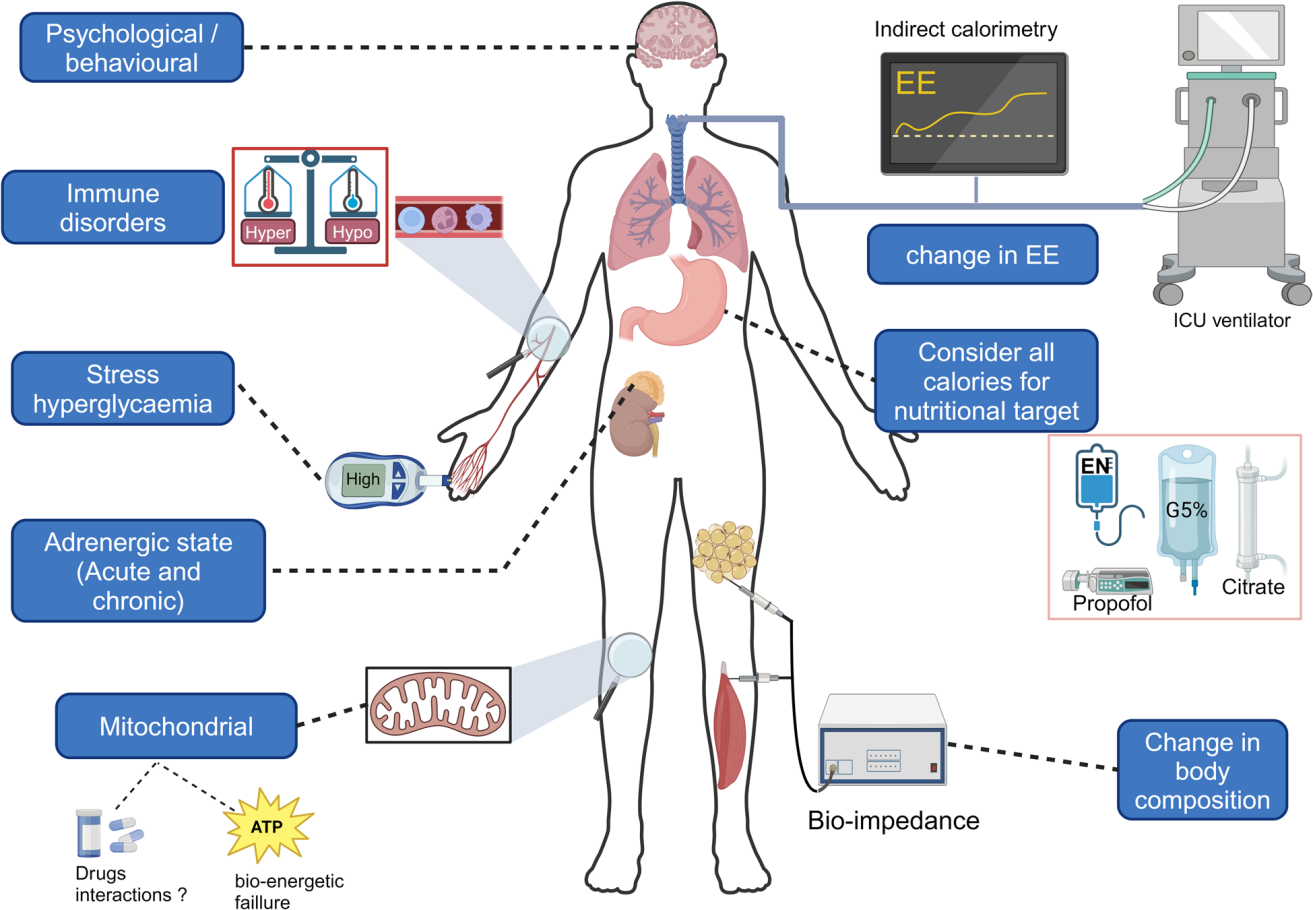
Overview of clinical consequences. The clinical consequences of the metabolic response to stress are varied and consist of: (1) Changes in energy expenditure measured by indirect calorimetry, with implications for risk of under- or over-nutrition, leading to inappropriate refeeding syndrome. All non-nutritional calories should be considered. (2) Stress hyperglycaemia. (3) Changes in body composition and the development of fat tissue and sarcopaenia, which can be measured by bioimpedance. (4) The development of states of hyper-adrenergism or cortisol deficiency depending on the phase of the metabolic stress response. (5) Immune disorders, (6) Mitochondrial dysfunction resulting in potential drug interactions and bioenergetic failure. (7) Behavioural changes and psychological sequelae. *EE* Energy Expenditure, *EN* Enteral Nutrition. *Created with BioRender.com*

In contrast to the section on pathophysiology and metabolic consequences, the clinical consequences and therapeutic implications can hardly be divided into acute, subacute, and chronic phases, since clinical manifestations may simultaneously affect all three phases, and numerous confounders can influence the clinical signs. The clinical consequences are therefore listed and discussed on the basis of Fig. 4.

### Changes in energy expenditure and requirements

EE varies across the acute, subacute, and chronic phases of the MRS. A slight initial increase in EE occurs during the acute phase, though it may be offset by sedation, opioids, or muscle paralysis, despite the heightened metabolic demands following the initial insult [8, 82]. This is followed by a more substantial rise in EE during the later phases [8].

Moreover, in the initial days, energy production from endogenous sources seems to increase due to a catabolic state, likely providing nearly enough energy to meet the body’s requirements. Full nutritional support during this phase may lead to overfeeding, as exogenous nutrition cannot fully suppress endogenous energy production, and the mitochondria are unable to process the excess substrate [53]. However, energy expenditure is reported to rise thereafter, remaining elevated for up to three weeks after ICU admission. During this period, endogenous energy production decreases, and exogenous nutritional support becomes essential. Indirect calorimetry remains the gold standard for accurately calculating energy expenditure at the bedside [5, 8, 11, 83, 84]. During the acute phase, provision of nutrition corresponding to 50–70% of the EE is recommended to prevent overfeeding or undernutrition, both of which are harmful [5, 9, 83, 85]. Even targeting 50–70% of energy expenditure might be excessive, regardless of the findings from the NUTRIREA-3 and EPANIC trials. The rationale for nutrition tailored to metabolic demands was not supported by the findings of a large-scale study on use of early parenteral nutrition [4]. A substudy [3] showed that early parenteral nutrition failed to prevent skeletal muscle wasting and increased adipose tissue in the muscle compartment without altering subcutaneous femoral or abdominal adipose tissue volume. Two retrospective studies [86, 87] suggested better survival rates when only 30–70% of energy needs were met during the initial ICU admission. NUTRIREA-3 trial [1] proposed a calorie restriction of 6 kcal kg^−1^ per day and 0.2–0.4 g kg^−1^ per day of protein during the first seven days, showing a faster readiness to discharge. Additionally, gradual administration of nutrition to meet targets helps prevent inappropriate refeeding syndrome in at-risk patients [83]. Restricted caloric intake during refeeding syndrome has been associated with an improvement in overall survival time [18]. In addition to nutrition, total calorie intake should include non-nutritional calories from glucose infusion or propofol and citrate during continuous renal replacement therapy [10].

After the acute phase, it is recommended that the amounts of energy and protein should be gradually increased to reach nutritional targets based on the phase of critical illness [5, 11, 83, 84]. However, nutritional therapy is not always effective when a state of anabolic resistance exists. The current challenge is to identify the right nutritional window and why, in some cases, nutrition fails to address the hypercatabolic state, despite following all current recommendations [20, 88]. The use of biomarkers and metabolomics studies is appealing because they could help to identify clinical endotypes of the MRS that have different therapeutic and nutritional needs depending on the phase of the stress response [7, 44, 73, 76, 89].

During all the MRS, the choice of nutritional route is crucial. Enteral nutrition is preferred, if possible, over parenteral nutrition because of its direct interaction with microbiota and the gut [5]. The clinical impact of dysregulation of the gut microbiome remains poorly elucidated with no current indications for one specific type of enteral nutrition over another or for pre or probiotic supplementation [90, 91]. Several gut-directed strategies are under investigation, including elemental or peptide-based formulas to reduce digestive workload, short-chain fatty acids such as butyrate for their anti-inflammatory effects and glutamine supplementation as a potential metabolic and immune support [40]. Additionally, glucagon-like peptide-1 (GLP-1) has been proposed to exert intestinotrophic effects, with recent data suggesting an association between higher GLP-1 levels and enhanced enterocyte recovery and reduced systemic inflammation in critical illness [92].

Enteral nutrition, however, carries risks of adverse effects, such as mesenteric ischaemia, especially in case of severe shock. Nevertheless, it is safe when carefully monitored and helps to prevent bacterial translocation as well as infectious complications [93].

There is growing interest in defining a fasting interval that could stimulate autophagy, mitochondrial biogenesis, and ketone production, though current evidence is limited to preclinical studies and remains unproven in ICU patients. [19, 94, 95].

Finally, the type of nutrient utilized could also influence MRS. For instance, omega-3 and omega-6 fatty acids play distinct roles in modulating inflammation, with omega-3 fatty acids contributing to the production of SPMs. Specific amino acids, such as arginine, leucine, and glutamine, may support immune function and promote recovery [89, 96]. The use of ketones or lactate as backup energy sources in acute situations is a promising field but there are currently no randomised controlled trials that support KBs production by fasting or dietary supplementation [19, 97, 98].

### Stress hyperglycaemia and glucose management

Stress-induced hyperglycaemia observed in 20 to 75% of cases, is associated with increased mortality [99], probably through detrimental increased oxidative stress [47]. This deleterious impact might however differ over time and from one patient to another, and controversy exists whether the relationship is causal or not. During this early phase, insulin resistance (IR) and associated stress hyperglycemia can be considered as a desirable mechanism of adaptation [61, 100–102]. Later on, IR can persist under the influence of several factors including persisting stress and inflammatory response, lipotoxicity due to the releasing of free fatty acids, treatments and prolonged immobilization [101–105]. Hence, IR can be considered as highly desirable when present during the first hours after the onset of stress, while on a long-term basis persistent IR can be viewed as detrimental [106], namely as a trigger of type 2 diabetes [107].

For the past 20 years, there has been ongoing debate regarding the benefits of tight glycaemic control. Despite promising effects of tight glucose control in pioneer randomized controlled trials [108], the benefit has not been confirmed in subsequent multicenter studies and one trial found potential harm [109]. Recently, TGC-Fast study [110] confirmed that intensive and tight glycaemic control managed by a computer algorithm to prevent iatrogenic hypoglycaemia, had no effect on the length of time that ICU care was needed or mortality when compared to a liberal strategy within the currently recommended nutritional doses [5]. Finally, a large patient level metaanalysis of 20 trials [111] found no advantages of intensive glucose control (risk ratio for mortality was 1.02 (95% CI, 0.96 to 1.07; P = 0.52).

In the interventional studies that have been conducted, patients have been treated uniformly, irrespective of diabetic status. However, observational studies [112] have suggested different associations of blood glucose levels with mortality in patients with and without pre-existing diabetes. To take this possible effect into account, the glycaemic ratio [113], which is calculated as the quotient of the mean blood glucose level during an ICU admission and the estimated average glucose prior to admission based on measured glycated haemoglobin A1 (HbA1c) values, has been used. In a retrospective study of more than 4500 ICU patients, the GR was associated with mortality, suggesting that patients with chronic hyperglycaemia may require different treatment than those without. Nevertheless, in the CONTROLING study [114], using a dynamic sliding scale insulin protocol, failed to confirm this strategy of individualized glucose control based on pre-admission usual glycemia. However, in this study the glycaemic correction algorithm generated too much hypoglycaemia (relative or absolute), which in itself is a factor associated with a poorer outcome [113, 115].

In light of the predominant use of glucose in the acute phase, some authors have proposed a more liberal glucose control of 180–252 mg dL^−1^, but although this approach was associated with fewer hypoglycaemic episodes, it had no effect on mortality and length of stay [116].

Latest guidelines recommend initiating protocols to treat persistent hyperglycaemia ≥ 180 mg dL-1 and targeting a range of 140–200 mg dL^−1^ to reduce the risk of hypoglycaemia [117].

### Changes in body composition and how to manage it ?

During critical illness, muscle loss occurs while adipose tissue increases [3, 118, 119]. All the available measurement techniques (bioimpedance, computed tomography, and ultrasound) show on average a 2% daily loss of muscle mass during the first week of ICU admission [120]. Body composition changes persist beyond ICU discharge: a lower lean mass and higher fat mass one year post-mechanical ventilation compared to controls [118]. Functionally, critically ill patients' adipose tissue enhances glucose and triglyceride storage [119]. ICU-AW, the result of muscle mass loss due to hypercatabolism and anabolic resistance, occurs in a large proportion of critically ill patients [121] and impacts morbidity and mortality. Limb weakness is independently associated with greater risk of extubation failure [122], which can prolong ICU stays thus potentially increasing long-term mortality rates [123].

Use of protein supplementation was initially suggested to counteract sarcopaenia in these patients. However, the most recent meta-analysis [124] which included 23 RCTs comparing higher versus lower protein supplementation strategies showed no improvement in survival, duration of ventilation, or hospital length of stay. In patients with acute kidney injury, higher protein delivery was associated with significantly increased mortality as well as the recent TARGET trial, which similarly suggested potential harm in patients with renal dysfunction [125]. Notably, the pooled daily protein intake in this meta-analysis was 1.49 ± 0.48 g.kg^−1^ for the high protein group versus 0.92 ± 0.30 g.kg^−1^ for the control group. Higher doses (≥ 2.2 g kg-1 day vs ≤ 1.2 g kg-1 day) were tested in 1,329 mechanically ventilated critically ill patients [126] in a pragmatic randomised trial, there was no overall effect on survival or hospital length of stay, although there was a negative signal in the subgroup of patients with acute kidney injury. Similarly, the PRECISe trial, a large double-blind, multicentre RCT including nearly 1000 ICU patients, found no benefit of high protein provision (2.0 versus 1.3 g·kg⁻^1^·day⁻^1^) on survival or functional recovery. On the contrary, patients in the high-protein group experienced a significant reduction in health-related quality of life at 180 days, together with a higher incidence of gastrointestinal intolerance. These findings reinforce concerns that higher protein strategies may not only fail to provide clinical benefit but could also adversely affect long-term outcomes in selected ICU populations [127].

Two phenomena could explain why protein supplementation may not be beneficial. Firstly, high ammonia levels from excessive protein supplementation may cause autophagy deficiency and mitochondrial toxicity [128, 129]. Secondly, anabolic resistance may be present, in which there is a reduced capacity to incorporate plasma amino acids into muscle protein synthesis, which is thus reduced despite normal protein absorption [130]. In addition, elevated glucagon levels, which can occur as a result of amino acid supplementation, have been implicated in increased amino acid catabolism, thus further limiting availability for protein synthesis [131]. The presence of anabolic resistance had been partly attributed to reduced mobility during the ICU stay. However, preliminary studies have suggested that early active mobilisation may be ineffective [132], or even associated with increased adverse events [133].

Anabolic agents such as hydroxymethylbutyrate and leucine show promise for improving the metabolic response to protein nutrition and countering muscle mass loss,but their efficacy in the general intensive care population requires further study [71, 134].

### Endocrine aspects and pharmacological modulation

Hormonal (mainly catecholamines) and inflammatory responses in critically ill patients cause increased workload on the cardiovascular and pulmonary systems to optimise oxygen delivery. A "hyper-adrenergic" state, favoured by administration of inotropic and vasopressor therapies, may also develop. High levels of catecholamines are associated with higher mortality [135]. The detrimental adrenergic state could be counteracted by giving beta-blockers [135]. Patients with traumatic brain injury treated with beta-blockers were shown to have improved survival in a meta-analysis [136], although the results should be interpreted cautiously due to the presence of bias. A randomised controlled trial (NCT01322048) is currently underway to confirm these results. Beta-blocker use has also been shown to improve neurological outcomes following cardiac arrest, and to improve wound healing, reduce the size of skin grafts in retrospective and RCT study [135] and improve hospital and 6-month mortality a post hoc analysis in burn patients [137].

Dysregulation of the hypothalamo-pituitary axis [23, 57] and tissue resistance to glucocorticoids puts patients at high risk of developing critical illness-related corticosteroid insufficiency. This condition may present as encephalopathy, delirium, fatigue, electrolyte disturbances, and persistent vasoplegia and may justify hydrocortisone treatment [26, 57].

RCT shows that supplementation with high-dose GH or thyroid hormone is generally considered detrimental in patients with hormonal deficiencies during the chronic phase [21, 23].

Exogenous angiotensin II and angiotensin 1–7 may have positive effects on septic shock, renal failure, and ARDS because of their immunomodulatory effects and impact on renal and pulmonary vasomotricity, respectively [27].

### Immune disorders and immune modulating agents

During sepsis, the inflammatory cascade has pro-and anti-inflammatory aspects. The intensity of the pro-inflammatory response conditions the intensity of the anti-inflammatory response [30, 31]. In 2016, SRS1 (hypoinflammatory) and SRS2 (hyperinflammatory) endotypes were identified in a genomic study [138] of 265 sepsis patients; the SRS1 endotype was associated with higher mortality (HR 2.4, 95% CI 1.3–4.5). These endotypes could distinguish two categories of death during sepsis [139]: early due to multiorgan failure and late (after 4 days), mainly from secondary infections likely associated with a hypoinflammatory subphenotype. More recently, an -omics approach has enabled the characterisation of sepsis into specific immune subphenotypes using high-dimensional technologies such as transcriptomics, proteomics, and metabolomics, identifying subtypes that reflect distinct immunological and pathophysiological characteristics [140]. In a transcriptomic analysis of over 1000 patients, three subphenotypes were identified and characterized by distinct immune-metabolic signatures and associated with different mortality risks. The Inflammopathic cluster showed high innate immune gene expression and was linked to early death; the Adaptive cluster exhibited lymphocyte-associated gene enrichment and better outcomes; the Coagulopathic group had altered coagulation and intermediate mortality [81].

Several therapies have been proposed without success [29, 141]. Only treatment with Corticosteroid supplementation is suggested in sepsis, ARDS, and severe community-acquired pneumonia (CAP) to reduce short-term mortality (14–30 days) with a variable strength of recommendation across guidelines [142, 143]. However, benefits may vary across subgroups, no consistent effect on long-term mortality has been shown, and a recent RCT reported no mortality benefit at all [144, 145]. A targeted therapy based on the inflammatory endotype (hypo or hyper) [30, 33] may improve outcome. For example, a low IFN (Interferon)-γ to IL-10 ratio has been proposed as a criterion for initiating hydrocortisone therapy in septic shock [146]. A placebo-controlled trial (NCT04280497) is currently underway to validate a biomarker-guided approach for initiating hydrocortisone therapy in this context. The efficacy of corticosteroids could be the result of relative endogenous glucocorticoid deficiency [57] and immunoregulatory effects [147]. The undesirable effects of corticosteroid use may include ICU-AW [144].

Furthermore, patients presenting with macrophage activation-like features or profound immunosuppression may respond differently to targeted immunotherapies, such as anakinra or interferon-γ, respectively [80].

### Bioenergetic failure or cytopathic hypoxia and therapeutic modulation

Mitochondrial dysfunction results in oxygen tissue utilisation or extraction abnormalities [148], which can be observed by the persistence of hyperlactataemia and/or PvaCO2xPavO2^−1^ ratio > 1 [149]. Their dysfunction also impacts autophagy and is directly linked to mortality. Furthermore, particularly in the liver, mitochondrial stress can down or up-regulate cytochrome enzymatic activity, impacting drug metabolism and potentially leading to over- or under-dosing of active metabolites [150]. The metabolic response may therefore affect the pharmacokinetics and pharmacodynamics of certain drugs by protein catabolism, and inflammatory cytokines lead to hypoalbuminaemia, affecting drugs that rely on free fraction or protein binding for their therapeutic effects. Drugs themselves can affect the metabolic response by altering EE [82] or directly impacting mitochondrial function [129, 151, 152].

Despite positive effects on mitochondrial function observed in preclinical models, no benefit on morbidity or mortality has been demonstrated with selenium or other antioxidants, and recent RCT even suggest potential harm with high-dose vitamin C in sepsis patients despite encouraging data in other small RCTs [153–157]. Zinc has been identified as beneficial in the burn patient population in terms of wound healing, with less requirement for regrafting [154]. Supplementation with thiamine is recommended in refeeding syndrome and in cases of suspected thiamine deficiency encephalopathy [158] and some retrospective studies support its administration in septic shock [159, 160]. Pharmacological activation of autophagy is an interesting approach but still at the preclinical stage [155].

### Behavioural changes and psychological sequelae after ICU and prevention strategies

Survivors of critical illness face risks of developing physical and psycho-cognitive disorders collectively termed "Post-Intensive Care Syndrome." The hypercatabolic state and secondary hyperglycaemia are associated with poorer long-term cognitive function [161]. Impairment of the hypothalamo-pituitary axis may contribute to the asthenia, delirium, anxiety, and sleep disturbances [21]. Various factors, including medication use, the disease itself, and painful stimuli, are associated with psychosocial and behavioural issues [161]. Recent rehabilitation guidelines [162] propose preventive strategies such as early mobilization and structured physical rehabilitation (Grade A), together with minimization of deep or prolonged sedation and daily interruption (Grade A). The ABCDEF bundle (Assessment, Breathing trials, Coordination of care, Delirium prevention, Early mobility, Family engagement) is proposed as a comprehensive approach (Grade A). Multidisciplinary post-ICU follow-up programs are suggested, although evidence remains limited (Grade C). Terapeutic implications and strategies are summarized in Table 2.

**Table 2:** Bedside management

Established and recommended	Possible	To be explored
– Target max 50–70% of the energy expenditure in the acute phase – Use indirect calorimetry to avoid over or under nutrition – Give enteral nutrition early, at least trophic nutrition – Monitor phosphate in patients at risk of inappropriate refeeding syndrome – Loose glycaemic control (140–200 mg/dL)	– Optimal glycaemic target may be personalised based on prior ICU glycaemic concentration (glycaemic ratio) – Micronutrient supplementation in at-risk or suspected deficient patients – Supplementation of ketones and lactate as rescue metabolic pathways – Timing: Intermittent feeding and fasting periods	– Assess subphenotypes of the metabolic response via continuous insulino-resistance monitoring, metabolomics and biomarkers to define metabolic profile and feeding responsiveness, – Targeting actors of the metabolic response regarding metabolic phases, with beta-blockers, antioxidants, or anti-inflammatory and anabolic agents – Gut-directed strategies and therapies

This table highlights the therapeutic implications associated with the metabolic response based on what is already established and recommended, what is possible, and what remains to be investigated

## Conclusion

The MRS is a complex condition involving various endocrine, neurohormonal, inflammatory, immune, oxidative and mitochondrial mechanisms. It impacts the entire human body and can influence the effects of commonly used medications in the ICU. A paradigm shift is gradually taking place around the concepts of energy targets through the various phases of MRS. There may be a narrow line between adaptive and maladaptive mechanisms, making it difficult to ensure a therapy provides benefit and not harm. Use of biomarkers and metabolomics should enable us to identify specific metabolic phases and clinical endotypes in which high protein ratios or different nutritional strategies, as well as anabolic, antioxidant or anti-inflammatory agents, may be beneficial.

## Supplementary Information

Additional file 1.

Additional file 2.

## Data Availability

The manuscript has no associated data.

## Abbreviations

ACTH: Adreno CorticoTropic Hormone
AMPK: AMP-activated protein kinase
ARDS: Acute respiratory distress syndrome
ATP: Adenosine triphosphate
AVP: Arginine vasopressin
BAT: Brown adipose tissue
BHB: β-Hydroxybutyrate
BMI: Body max index
CAP: Community-acquired pneumonia
CCI: Chronic critical illness
DAMPs: Damage-associated molecular patterns
EE: Energy expenditure
FSH: Follicle-Stimulating Hormone
FFA: Free fatty acid
GH: Growth hormone
GLP: Glucagon-like peptide
GLUT: Glucose transporter
ICU: Intensive care unit
ICU-AW: Intensive care unit-acquired weakness
IL: Interleukine
IFN-γ: Interferon gamma
IMC: Intermediate care.
KBs: Ketone bodies
MRS: Metabolic response to stress
mTOR: Mammalian target of rapamycin
NLRP3 inflammasome: NOD-like receptor family, pyrin domain containing 3
PAMPs: Pathogen-associated molecular patterns
PI3K: Phosphatidylinositol 3-kinase
PICS: In this article, the acronym PICS has been chosen to replace the “Persistent Inflammation, Immunosuppression, and Catabolism Syndrome”encountered in the chronic phase of the metabolic stress response. It should not be confused with the same acronym PICS, which stands for Post-Intensive Care Syndrome (abbreviation not present in this article but frequently found in the literature)
PRL: Prolactin
RCT: Randomized Controlled Trial
ROS: Reactive oxygen species
SCFA: Short-chain fatty acids
SPM: Specialized Pro-resolving Mediators
TLR: Toll-like receptors
TNF: Tumour necrosis factor
WAT: White adipose tissue
