# Supplementary material for: The metabolic response to stress in critical illness: updated review on the pathophysiological mechanisms, consequences, and therapeutic implications
Source: Ann Intensive Care. 2025 Oct 27;15:174. doi: 10.1186/s13613-025-01588-z (PMC12554859; doi:10.1186/s13613-025-01588-z)
Supplement: Supplementary file 1 — Additional file 1. [file 13613_2025_1588_MOESM1_ESM.docx]

**Annexe 1 : Glossary of Specialized Terms in Metabolic Stress Response during Critical Illness**

| Term | Definition |
| --- | --- |
| Phenotype    Subphenotype  Endotype | A clinically observable set of clinical features; syndromes such as acute respiratory distress syndrome (ARDS) and sepsis could be considered as phenotypes.  a distinct subgroup of a phenotype based on a shared set or pattern of observable or measurable properties, which can be reliably discriminated from other subphenotypes.  a subphenotype with distinct functional or pathobiological mechanisms that may or may not be associated with a specific treatment response.  **Phenotyping terminology is based on consensus Report and Recommendations (Gordon, AMJRCC 2024. PMID: 38687499)** |
| Anabolic resistance | Reduced ability of tissues (mainly muscle) to respond to anabolic stimuli, impairing protein synthesis despite adequate nutrient supply. |
| Autophagy | Cellular self-digestion mechanism that recycles damaged or unnecessary components. Crucial during stress for metabolic adaptation and survival. |
| Bioelectrical Impedance Analysis (BIA) | A non-invasive method to assess body composition by measuring the resistance and reactance of body tissues to a low-intensity electrical current. BIA provides estimates of fat mass, lean mass, and total body water |
| Bioenergetic failure | Failure of cellular energy production, mainly due to mitochondrial dysfunction |
| Chronic critical illness (CCI) | A condition defined by prolonged ICU stay (typically >14 days) accompanied by persistent organ dysfunction, dependence on life support therapies, and features of catabolism, immune suppression, and neurocognitive impairment. It often overlaps with PICS and results in high long-term morbidity and mortality. |
| Energy Expenditure (EE) | The total amount of energy used by the body to maintain essential physiological functions and respond to stress. In critically ill patients, EE is influenced by inflammation, hormonal changes, and organ dysfunction. It can be measured directly via indirect calorimetry or estimated using predictive equations. |
| Enterohormones | Hormones secreted by the gut (e.g., GLP-1, PYY, ghrelin) that regulate gastric emptying, appetite, absorption, and nutrient metabolism. |
| Glycaemic ratio | Ratio of average ICU glucose to estimated pre-admission glucose (from HbA1c) |
| Indirect calorimetry | Technique measuring energy expenditure via gas exchange (O₂/CO₂), used to guide ICU nutritional support. |
| Insulin resistance | Decreased sensitivity of cells to insulin, leading to inefficient glucose use and hyperglycaemia, especially heightened during critical stress. |
| Ketone bodies | Energy substrates (e.g., β-hydroxybutyrate) produced by the liver during fasting or stress, serving as an alternative to glucose. |
| Lactormone | A term highlighting the signaling role of lactate beyond its metabolic function. Lactate acts as an autocrine, paracrine, and endocrine-like signaling molecule, modulating immune responses, cellular communication, and metabolic regulation. |
| Metabolic shift (during acute injury) | Transition in energy production, from mitochondrial β-oxidation of fatty acids toward glycolysis, branched-chain amino acid metabolism, and activation of the pentose phosphate pathway |
| Mitochondrial fusion and fission | Dynamic reshaping mechanisms of mitochondria. Their imbalance contributes to mitochondrial dysfunction seen in ICU patients. |
| Mitophagy | A specific type of autophagy targeting dysfunctional mitochondria, essential for maintaining cellular energy homeostasis. |
| mTOR | Key protein in cellular signaling that regulates growth and protein synthesis; inhibited during stress to enhance autophagy. |
| NETosis | Process by which neutrophils release extracellular traps (NETs) to capture pathogens. |
| Non-nutritional calories | Caloric input from medications or solutions (e.g., propofol, citrate, IV glucose) |
| Persistent Inflammation, Immunosuppression, and Catabolism Syndrome (PICS) | A clinical syndrome characterized by ongoing systemic inflammation, immune dysfunction, and muscle wasting after the acute phase of critical illness |
| Pituitary factors | Hormones secreted by the pituitary gland (e.g., GH, ACTH) that regulate metabolic and endocrine responses to stress. Their secretion may be altered in critical illness. |
| Pro-catabolic hormones | Hormones (e.g., cortisol, glucagon,catecholamines) that promote tissue breakdown to release energy substrates. |
| Refeeding syndrome | A potentially life-threatening condition that occurs upon reintroduction of nutrition after a period of prolonged fasting or undernutrition. It is characterized by acute shifts in fluids and electrolytes, particularly hypophosphatemia, hypokalemia, and hypomagnesemia, resulting from increased insulin secretion and cellular uptake. |
| SPMs | Specialized Pro-resolving Mediators derived from omega-3 fatty acids that promote the active resolution of inflammation. |
| Stress hyperglycaemia | Transient high blood glucose due to acute physiological stress, independent of pre-existing diabetes. |
