# Supplementary material for: The metabolic response to stress in critical illness: updated review on the pathophysiological mechanisms, consequences, and therapeutic implications
Source: Ann Intensive Care. 2025 Oct 27;15:174. doi: 10.1186/s13613-025-01588-z (PMC12554859; doi:10.1186/s13613-025-01588-z)
Supplement: Supplementary file 2 — Additional file 2. [file 13613_2025_1588_MOESM2_ESM.pdf]

SUPPLEMENTARY MATERIAL - SEARCH CRITERIA AND SYNTHESIS :

For this narrative review, given the broad nature of the topic, we first developed a general search equation to identify all fundamental concepts inherently linked to the metabolic response to stress. We initially searched for English-language publications in the PubMed database from its inception until December 2024. The search equation used was: "((metabolism[MeSH Terms]) OR (metabolic stress response[MeSH Terms])) AND (critical illness[MeSH Terms])" (n = 2,877).

Since our review is based on concepts that each warrant a dedicated literature review, we identified fundamental concepts (or keywords) related to the metabolic response to stress in critically ill patients. These fundamental concepts were determined by consensus among the authors based on the retrieved articles. Each of these fundamental concepts was then used for an additional PubMed search, covering the period from January 2014 (the year of the last major review on this topic) to December 2024. This secondary search focused primarily on review and synthesis articles to extract the most recent insights.

For pathophysiology, the fundamental concepts identified were: "endocrine response," "inflammation OR cytokines," "gastrointestinal tract OR microbiota," "mitochondrial dysfunction," "reactive oxygen species," "autophagy," "catabolism," "anabolic resistance," and "metabolomics."

For metabolic and clinical consequences, the fundamental concepts were: "stress hyperglycaemia," "insulin resistance," "energy substrate," "energy expenditure," "body composition," "nutritional support," "post-intensive care syndrome," and "persistent inflammatory catabolic syndrome."
